# Investigation of Fellowship Leadership in Orthopaedic Musculoskeletal Oncology

**DOI:** 10.5435/JAAOSGlobal-D-22-00039

**Published:** 2022-06-10

**Authors:** M. Lane Moore, Muhammad Ali Elahi, Matthew K. Doan, Jordan R. Pollock, Justin L. Makovicka, Jeffrey D. Hassebrock, Joseph C. Brinkman, Karan A. Patel

**Affiliations:** From the Mayo Clinic Alix School of Medicine, Scottsdale, AZ (Mr. Moore, Mr. Elahi, Mr. Doan, and Mr. Pollock), and the Department of Orthopedic Surgery, Mayo Clinic, Phoenix, AZ (Dr. Makovicka, Dr. Hassebrock, Dr. Brinkman, and Dr. Patel).

## Abstract

**Introduction:**

The purpose of this study was to determine the objective characteristics of orthopaedic musculoskeletal oncology fellowship directors (FDs) by concentrating on the demographics, academic background, institutional history, research experience, and professional affiliations of these leaders.

**Methods:**

Data were collected for each FD through institutional biographies or publicly available curriculum vitae. The data collected for each FD included demographic, professional, and research information.

**Results:**

Of the 19 FDs, 15 (78.9%) were male, and 4 (21.1%) were female. The mean age for all FDs was 49.2 ± 9.1 years. Most FDs were White (n = 16; 84.2%). The mean Scopus H-index, total number of citations, and total number of publications among all 19 FDs were 21.6 ± 13.8, 2,290.6 ± 2,709.0, and 84.0 ± 54.7, respectively. The mean number of years serving in the FD role was 7.1 ± 9.1 years, and the mean number of years that the FD was employed at his/her current institution was 11.1 ± 8.1 years.

**Conclusion:**

This study shows that orthopaedic musculoskeletal oncology FDs were mainly White (84.2%), male (78.9%), and in their late 40s; have filled their role as FD for an average of 7.1 years; and are very productive in research.

Over the past several decades, the field of orthopaedic surgery has seen an increasing rate of surgeon specialization. During this period, the United States has seen a growing number of residency-trained orthopaedic surgeons seeking additional fellowship training in various subspecialty areas.^1-3^ A recent study estimated that 76% of orthopaedic surgeons seeking board certification in 2003 completed some form of fellowship training, and this percentage increased to 90% in 2013.^4^ With an increasing number of orthopaedic residents seeking fellowship training, the influence of fellowship directors (FDs) has an increasingly notable impact on future orthopaedic surgeons. FDs are typically leaders in their respective fields and often possess unique skillsets and achievements that deserve recognition.

The trend toward subspecialization in orthopaedic surgery began in the 1980s when the Accreditation Council for Graduate Medical Education established accredited orthopaedic fellowship training programs. However, the pathway for orthopaedic musculoskeletal oncology fellowship training was formalized in a slightly different manner with the establishment of the Musculoskeletal Tumor Society (MSTS) in 1977.^5^ Formerly, the territory of general surgeons, sarcoma treatment, and subsequent reconstruction was a primary focus of the orthopaedic surgeons in the MSTS. Advances in chemotherapy, radiation oncology, imaging, surgical techniques, and reconstructive prosthetics and implants brought orthopaedic physicians to the forefront of sarcoma treatment.^5,6^ With this advancement came the establishment of orthopaedic oncology training programs throughout the United States and the further development of the field of orthopaedic oncology into a unique field within orthopaedic surgery.^7^ Currently, surgeons practicing within this field may further specialize in adult or pediatric orthopaedic oncology. In addition, musculoskeletal orthopaedic surgeons may further subspecialize their practice based on the body location (eg, orthopaedic surgeons completing a spine and orthopaedic oncology fellowship to treat spinal pathologies) or pathology (eg, specializing in the treatment of sarcomas, intra-articular tumors, and reconstructive surgery).^8^

Many previous research studies in the field of orthopaedic residency and fellowship programs have focused on factors that determine a successful applicant. Such studies have examined topics such as orthopaedic applicant characteristics, factors influencing students to choose a career in orthopaedics, and selection criteria for a wide pool of fellowship applicants.^2,9,10,11,12,13,14^ However, few studies have attempted to investigate topics relating to orthopaedic fellowship leadership, such as factors that make for a successful career as a leader in orthopaedic residency and fellowship programs. Within the field of orthopaedics, a handful of studies have sought to describe the leadership characteristics within spine surgery, sports medicine, foot and ankle surgery, and adult reconstructive surgery FDs, respectively.^15,16,17,18^ However, no previous publication has examined the leadership qualities among orthopaedic musculoskeletal oncology FDs.

Orthopaedic musculoskeletal oncology FDs are known to be accomplished physicians who possess a broad array of leadership, academic, professional, and clinical achievements. However, it is unknown what objective qualities set these capable surgeons apart from their peers. Therefore, the purpose of this study was to determine the objective characteristics of orthopaedic musculoskeletal oncology FDs by concentrating on the demographics, academic background, institutional history, research experience, and professional affiliations of these leaders.

## Methods

The MSTS Orthopaedic Musculoskeletal Oncology Fellowship Listing for 2020 to 2021 was queried to compile a list of all Accreditation Council for Graduate Medical Education–accredited orthopaedic oncology fellowships (accessed on December 2020).^19^ Only fellowships in the United States were included in this analysis. The listing from the MSTS website was cross-referenced with the SF Match 2020 orthopaedic musculoskeletal oncology fellowship listing to ensure accuracy and consistency.^20^ The FD and fellowship program coordinator/administrator were identified from the MSTS and SF Match websites. Demographic, educational, and professional background data were collected for each FD by reviewing institutional biographies, personal websites, and publicly available curriculum vitae (CVs). If all desired variables were unable to be collected after careful review of publicly available resources, an electronically mailed (e-mailed) questionnaire was sent to the FD or their fellowship program coordinator/administrator who requested the missing data points. If there was no response to the initial e-mailed questionnaire, a follow-up e-mail and/or phone call were made.

Data collected for each orthopaedic musculoskeletal oncology FD included the following: age, sex, race/ethnicity, past medical school program, past residency training location, past fellowship training location, residency and fellowship graduation years, additional advanced degrees, military affiliation, institutional loyalty, year hired by current institution, time since residency and fellowship completion until FD appointment, and length of time in the FD role. Furthermore, each FD's H-index, total number of publications, and total number of citations were collected in an effort to measure research productivity and impact.

The H-index metric is an estimation of an individual's scientific productivity and impact. H-index is defined as the maximum value of *h* such that the author in question has published *h* scientific manuscripts that have all been cited a minimum of *h* times.^21^ For example, an author with an H-index of 15 must have produced at least 15 scientific, peer-reviewed manuscripts with at least 15 citations each. The H-index metric, total number of publications, and total number of citations were all obtained from the Scopus database by searching the FDs first and last name (Elsevier BV).^22^ The Scopus database is composed of a vast record of peer-reviewed scientific literature that automatically tabulates metrics such as H-index, total number of publications, and total number of citations for the authors. A publication was defined as any piece of peer-reviewed scientific literature where the author's name was credited anywhere on the author line. The total number of citations was calculated by the Scopus database by tabulating all the instances where an author was acknowledged for his or her scientific works.

Statistical analysis involved the calculation of Pearson correlation coefficients. Statistical analysis was performed using Microsoft Excel (Microsoft Corp). The correlation coefficients calculated in this analysis were interpreted according to Mukaka's^23^ guide on correlation coefficient interpretation in medical research settings. Correlation coefficient values <0.4, 0.4 to 0.7, 0.7 to 0.9, and >0.9 are suggestive of weak, moderate, strong, and very strong positive correlation, respectively.^24^

## Results

**Figure 1:**
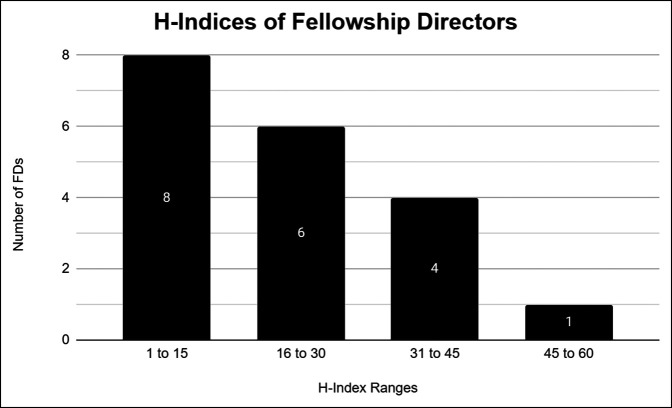
H-indices of orthopaedic musculoskeletal oncology fellowship directors (FDs).

According to the MSTS Orthopaedic Musculoskeletal Oncology Fellowship Listing for 2020 to 2021 and the SF Match 2020 orthopaedic musculoskeletal oncology fellowship listing, there were a total of 19 accredited orthopaedic musculoskeletal oncology fellowship programs in the United States and 19 individual FDs. Appropriate data were collected for all 19 musculoskeletal oncology FDs included in the listings. Overall, 15 FDs were male (78.9%), and 4 FDs were female (21.1%). The mean age was 49.2 ± 9.1 years. In total, six additional advanced degrees were held between five (26.3%) FDs. This included one PhD, one MPH, and four MS/MA's. One FD had both a PhD and an MA. No FDs were found to have a known military affiliation. The mean Scopus H-index, total number of citations, and total number of publications among all 19 FDs were 21.6 ± 13.8, 2,290.6 ± 2,709.0, and 84.0 ± 54.7, respectively. Figure 1 stratifies the H-indices of all FDs. In addition, the 10 orthopaedic musculoskeletal oncology FDs with the highest H-indices are included in Table 1 along with their total number of publications and total number of citations. In regard to race and ethnicity, most FDs were White (n = 16; 84.2%), followed by Black or African American (n = 2; 10.5%) and Asian (n = 1; 5.3%).

**Table 1:** Research Metrics for the 10 Most Prolific Orthopaedic Musculoskeletal Oncology Fellowship Directors as Determined by the Scopus H-Index, Total Number of Publications, and Total Number of Citations

Orthopaedic Oncology Fellowship Leader Research Productivity				
FD Name	H-Index	Total No. of Publications	Total No. of Citations	Fellowship Program Name
Rex Haydon, MD, PhD	56	138	10,539	University of Chicago Musculoskeletal Oncology
Ernest Conrad, MD	42	122	6,920	McGovern Medical School Orthopaedic Oncology Fellowship
R. Lor Randall, MD, FACS	35	200	5,162	Sarcoma Advanced Research and Clinical Fellowship—Musculoskeletal Oncology
Mark Scarborough, MD	34	126	3,595	University of Florida Musculoskeletal Oncology Fellowship
Valerae O. Lewis, MD	33	125	3,203	UT M.D. Anderson Cancer Center Musculoskeletal Oncology
Kevin Jones, MD	26	111	2,558	University of Utah/Huntsman Cancer Institute—Musculoskeletal Oncology Fellowship
Matthew Houdek, MD	24	159	1,610	Mayo Musculoskeletal Oncology Fellowship
Kevin Raskin, MD	23	92	2,031	Massachusetts General Hospital Fellowship-Tumor
Ginger Holt, MD	23	92	1,520	Vanderbilt Orthopaedic Institute Musculoskeletal Oncology
Nicholas Bernthal, MD	20	101	1,330	UCLA Musculoskeletal Oncology Fellowship

FD = fellowship director

The Scopus H-index values and total number of citations are as of December 2020.

When analyzing education and employment timeline, the mean calendar year for residency and fellowship graduation was 2004 ± 8.9 and 2006 ± 8.8, respectively. In addition, the mean number of years from fellowship graduation to appointment as FD was 7.7 ± 8.8 years. Among the cohort sampled, the mean number of years serving in the FD role was 7.1 ± 9.1 years, and the mean number of years that the FD was employed at his/her current institution was 11.1 ± 8.1 years. On average, a FD was appointed to the role of FD 4.6 ± 3.8 years after their year of hire.

When looking at institutional loyalty, three FDs (15.8%) were currently serving at or affiliated with the same institution in which they attended medical school. Furthermore, five FDs (26.3%) were affiliated with the same institution or hospital where they completed residency, and six FDs (31.6%) were affiliated with the same program in which they completed fellowship. All demographic, educational, and training data are summarized in Tables 2 and 3.

**Table 2:** Demographics, Training Background, Education and Employment Progression, and Leadership Positions of Orthopaedic Musculoskeletal Oncology Fellowship Directors

Overall leadership	N (%)
Total no. of fellowship programs	19
Total no. of fellowship directors	19
Demographics	N (%) or mean score ± SD
Male	15 (78.9)
Female	4 (21.1)
Mean age (yr)	49.2 ± 9.1 (n = 15)
Advanced degrees	N (%)
PhD	1 (5.3)
MBA	0 (0.0)
MPH	1 (5.3)
MS/MA	4 (21.0)
Training and research	N (%) or mean score ± SD
Military affiliation	0 (0.0)
Mean FD Scopus H-index	21.6 ± 13.8 (n = 19)
Mean no. of total citations	2,290.6 ± 2,709.0 (n = 19)
Mean no. of publications	84.0 ± 54.7 (n = 19)
Race/ethnicity	N (%)
American Indian or Alaskan Native	0 (0.0)
Asian	1 (5.3)
Black or African American	2 (10.5)
Hispanic or Latino	0 (0.0)
Native Hawaiian or other Pacific Islander	0 (0.0)
White	16 (84.2)
Education and employment progression	Mean score ± SD
Mean residency graduation calendar year	2004 ± 8.9 (n = 17)
Mean fellowship graduation calendar year	2006 ± 8.8 (n = 18)
Mean no. of years from fellowship graduation to FD appointment	7.7 ± 8.8 (n = 11)
Mean no. of years of FD employment at his/her current institution	11.1 ± 8.1 (n = 16)
Mean no. of years in the FD role	7.1 ± 9.1 (n = 11)
Mean no. of years from year of hire to year appointed FD	4.6 ± 3.8 (n = 11)
Institutional loyalty	N (%)
FDs currently working at same institution as medical school graduation	3 (15.8)
FDs currently working at same institution as residency graduation	5 (26.3)
FDs currently working at same institution as fellowship graduation	6 (31.6)
Correlated H-indices	r (*P* value)
Years as FD vs Scopus H-index	0.43 (0.18)
Age vs Scopus H-index	0.64 (0.01)^a^

FD = fellowship director

a Indicates that the correlation is significant

**Table 3:** Sex and Racial Breakdown of the US Medical Student, Orthopaedic Resident, Orthopaedic Physician, and Musculoskeletal Oncology FD Cohorts as of 2019^34,35^

Factor	US Cohort	US Medical Students	US Orthopaedic Residents	AAOS Membership	Musculoskeletal Oncology FD
White (%)	60.7	56.0	75.6	86.8	84.2
African American (%)	13.4	5.7	4.0	1.5	10.5
Hispanic/Latino (%)	18.1	5.4	5.4	1.7	0.0
Asian American (%)	5.8	21.0	13.7	6.7	5.3
Native American (%)	1.3	0.1	0.2	0.4	0.0
Female (%)	51.1	50.5	15.4	5.0	21.1

AAOS = American Academy of Orthopaedic Surgeons, FD = fellowship director

Interestingly, no single medical school, residency program, or fellowship program was overwhelmingly represented among orthopaedic musculoskeletal FDs. With regard to medical schools, Yale University School of Medicine and Northeastern Ohio Universities College of Medicine graduated the most future FDs (n = 2 each), whereas all 15 other FDs attended different medical schools. Only the UCLA medical center's orthopaedic residency program graduated more than one future FD (n = 2). Finally, only Massachusetts General Hospital (n = 2), Mount Sinai Hospital (n = 2), the University of Washington (n = 3), the University of Chicago (n = 2), and the University of Florida College of Medicine (n = 2) orthopaedic musculoskeletal oncology fellowship program graduated more than one future FD. The complete list of medical school, residency, and fellowship training programs of current FDs is included in Table 4.

**Table 4:** Previously Attended Medical Schools, Residencies, and Fellowship Training Programs of the Currently Analyzed Orthopaedic Musculoskeletal Fellowship Directors

Medical School (No. Attended)	Residency Training Program(No. Attended)	Fellowship Training Program(No. Attended)
Beijing Medical University (1)	Harvard Combined Orthopaedic Residency Program (1)	Moffitt Cancer Center (1)
Columbia University, College of Physicians and Surgeons (1)	Hospital for Special Surgery (1)	Huntsman Cancer Institute (1)
Harvard Medical School (1)	Mayo Graduate School of Medicine (1)	International Center for Limb Lengthening (1)
Johns Hopkins University (1)	Monmouth Medical Center (1)	Massachusetts General and Boston Children's Hospital (2)
Northeastern Ohio Universities College of Medicine (2)	Montefiore Hospital & Medical Center (1)	Memorial Sloan Kettering Cancer Center (1)
Tel-Aviv University School of Medicine (1)	New York Presbyterian/Columbia University (1)	Mount Sinai Hospital (2)
Temple University School of Medicine (1)	Peking University People's Hospital, University of Texas (1)	The University of Washington Medical Center (3)
Tufts University School of Medicine (1)	Summa Health System (1)	The University of Chicago (2)
University of Alabama at Birmingham (1)	University of California San Francisco (1)	University of Florida College of Medicine (2)
University of Arizona College of Medicine (1)	University of California Los Angeles Medical Center (2)	University of Medicine and Dentistry of New Jersey (1)
University of Chicago Pritzker School of Medicine (1)	University of Chicago Medicine (1)	University of Miami Department of Orthopaedics (1)
University of Florida College of Medicine (1)	University of Iowa Hospitals and Clinics (1)	University of Colorado Hospital (1)
University of Kansas School of Medicine (1)	University of Medicine and Dentistry of New Jersey (1)	Washington Cancer Institute at Washington Hospital Center (1)
University of South Florida (1)	University of Minnesota Medical School (1)	—
University of Virginia (1)	University of South Florida (1)	—
Weill Cornell Medical College (1)	University of Texas Medical Branch at Galveston (1)	—
Yale University Medical School (2)	Vanderbilt University Medical Center (1)	—
—	Virginia Commonwealth University (1)	—

The calculation of Pearson correlation coefficients discovered a statistically significant moderate correlation between age and Scopus H-index (r = 0.64; *P* = 0.01). However, the relationship between years as FD and Scopus H-index was not identified to be a statistically significant correlation (r = 0.43; *P* = 0.18).

## Discussion

The present analysis discovered that most orthopaedic musculoskeletal oncology FDs are White (84.2%), male (78.9%), and in their late 40s; have no additional advanced degrees (73.7%); have extensive research credentials and accomplishments, and have filled their role as FD for an average of 7.1 years. In addition, the level of research aptitude and achievement among orthopaedic oncology FDs is of note. The mean Scopus H-index across all 19 FDs was 21.6, with 57.9% of FDs having an H-index of over 15. The average number of citations and publications was 2,290.6 and 84.0, respectively. This equates to 1,596 total publications and 43,522 total citations between the 19 FDs included in this analysis. This impressive achievement of orthopaedic oncology FDs may be one factor that has contributed to the attainment of such prestigious positions.

To provide more context for the high level of research accomplishment achieved by the FDs in this study, similar analyses performed in orthopaedic/neurologic spine surgery, orthopaedic adult reconstructive surgery, and orthopaedic trauma surgery found that FDs had an average H-index of 23.8, 16.5, and 15.1, respectively.^15,18,25^ This would place orthopaedic musculoskeletal oncology surgeons just behind spine surgeons as the most prolific researchers and scientists within the field of orthopaedics. Furthermore, a study by Bastian et al^26^ discovered that the mean H-index score among 2,061 academic orthopaedic surgeons with no specified subspecialty who held chair positions within their respective institutions was approximately 17.8. Therefore, it is likely that the field of orthopaedic musculoskeletal oncology places a high value on research production and favors physicians who have proven themselves to be not only skillful surgeons but also productive research scientists. The field of orthopaedic musculoskeletal oncology also benefits from the fact that it sits at the intersection of two fields highly active in research. Both orthopaedic surgeons and oncologists treat and refer patients with bone cancers. Therefore, this field of musculoskeletal oncology draws attention and citation generation from multiple disciplines and training backgrounds. Moreover, research plays an especially valuable and important role in the field of musculoskeletal oncology because the field works to continuously improve its treatment and procedure options while also expanding the patient cohort that it can treat.^27^

The orthopaedic musculoskeletal oncology FDs included in this study received previous medical, orthopaedic, and oncological training at a wide variety of institutions (18 different residency programs and 13 different fellowship programs). This is in stark contrast to previous studies performed in orthopaedic/neurologic spine and orthopaedic adult reconstructive FDs, where FDs often come from a small handful of training programs.^15,18^ In the previous analysis in spine surgeons by Donnally et al., 19 FDs (18%) received training from only four orthopaedic residency programs. In addition, 32 FDs (31.1%) were trained at only four fellowship programs.^15^ This trend is also present in adult reconstructive surgery, where six orthopaedic residency programs and eight adult reconstructive fellowship programs produce 20 (21.3%) and 43 (45.7%) FDs, respectively.^18^ It is often assumed that certain training programs have the potential to offer trainees additional access and opportunities to career advancement, mentorship, and professional networking. In academic medicine, hierarchy and networking play an important role in career advancement.^28-30^ This seems to be the case in orthopaedic fields such as spine surgery and adult reconstructive surgery. However, the findings from this study of orthopaedic oncology FDs suggest that the educational pedigree of orthopaedic musculoskeletal oncology FDs may play less of a role in leadership position acquisition.

In terms of educational and orthopaedic training timelines, musculoskeletal oncology FDs seem to be similar but slightly more accelerated than similar fields. For example, the mean year of residency and fellowship graduation in this study was 2004 and 2006, respectively. This is in comparison with mean residency and fellowship graduation years of 1999 and 2001 for spine and 2000 and 2001 for adult reconstruction.^15,18^ In addition, the mean number of years between fellowship graduation and FD appointment in this study was 7.7 years compared with 9.6 and 8.6 years in adult reconstruction and spine, respectively. The average orthopaedic musculoskeletal oncology FD was appointed to their leadership role 4.6 years after they were hired at their current institution. Spine and adult reconstructive surgery FDs had mean year of hire to year appointed FD intervals of 4.7 and 5.5, respectively.^15,18^ Finally, the mean number of years serving in the FD role was 7.1 years for oncology, 8.2 years for adult reconstruction, and 9.7 years for spine.^15,18^ In summary, these findings suggest that orthopaedic musculoskeletal oncology FDs are younger (average age of 49.2 compared with 52.8 in spine and 52.6 in adult reconstruction) and experience slightly accelerated timelines in terms of advancement to the role of FD. The causation for such a trend is unclear and may warrant future investigation.

We noted a lack of racial, ethnic, and sex diversity among orthopaedic musculoskeletal oncology FDs. Most FDs included in this analysis were White males in their late 40s. Only four FDs were female (21.1%), and only three FDs were non-White (15.8%). These findings are consistent with the field of orthopaedic surgery as a whole.^31-33^ However, to put these findings in additional context, it is important to compare the female and minority makeup of the musculoskeletal oncology field with the broader US cohort and physician workforce. In 2019, approximately 60.7% of Americans were White, whereas 13.4% were African American, 18.1% were Hispanic, 5.8% were Asian American, and 1.3% were Native American.^34^ Compared with the entire US cohort, the field of musculoskeletal oncology is somewhat similar because they have comparable representation of African American and Asian individuals. However, Hispanic Americans are severely underrepresented, and White Americans are overrepresented.

In addition, compared with the cohort of US orthopaedic surgeons, the field of musculoskeletal oncology has a similar racial breakdown. In 2019, 86.6% of orthopaedic residents were White, whereas 1.5% were African American, 1.7% were Hispanic, 6.7% were Asian, and 0.4% were Native American.^34^ Therefore, compared with the entire field of orthopaedic surgery, musculoskeletal oncology is better representative of African Americans and is consistent with the representation of White and Asian orthopaedic surgeons.

Orthopaedic surgery has long been a male-dominated surgical subspecialty and only recently has the field seen a rise in the representation of females and minority physicians. As of 2017, approximately 5.0% of all orthopaedic surgeons in the United States were female.^35^ Over the past two decades, the percentage of female orthopaedic residents has risen moderately from 11.0% in 2005 to 14% in 2017 (+27.3% increase).^29,35,36^ Interestingly, the field of musculoskeletal oncology is more representative of female surgeons (21.1%) than the orthopaedic workforce as a whole (5.0%).^35^

However, the field of orthopaedics still lags behind other fields in the rate of increased female and minority representation.^29,37^ Conversely, the field of orthopaedic surgery has actually seen a decline in minority representation within residency training programs since 2006. Poon et al^36^ report a decline of 32% in minority representation between 2006 (33.3%) and 2015 (22.5%). Unfortunately, more granular data representing trends within the cohort of FDs are not available. This finding is important to note because the greatest perceived barrier to increasing racial and ethnic diversity within the field of orthopaedics is the already present lack of underrepresented minority faculty and underrepresented minorities in leadership positions.^38^

Previous research has shown that minority physicians are more likely to provide care for minority patient cohorts and display an increased level of cultural competence when treating these cohorts.^39,40^ Therefore, further representation of minorities and women in orthopaedic oncology and leadership could be beneficial for improving patient care throughout the field. Compared with similar fields such as spine surgery and adult reconstructive surgery, musculoskeletal oncology had a larger proportion of female FDs (3.9% in spine and 0.0% in adult reconstruction). However, much work still needs to be done to improve the female and minority representation across the field of orthopaedic surgery.

This study has several limitations. First, the acquisition of FD data for this study relied on self-reported and potentially inaccurate information in the form of website biographies, CV, and e-mail questionnaires. It is a possibility that pieces of information reported online or in CVs are inaccurate or outdated. However, the authors responsible for collecting the FD data in this study were careful to corroborate pieces of data from multiple online sources and used direct e-mail questionnaires when ambiguity was noted, or inaccuracy was suspected. Second, contacting FDs or their fellowship program administrative staff was not always successful. As a result, data for a handful of FDs were only partially completed. In addition, this analysis represents only a single point in time. FD appointments often change from year to year, and new fellowship programs are frequently started. Finally, many important characteristics of a successful orthopaedic surgeon and FD were not able to be collected. For example, surgical skill, communication proficiency, and bedside manner are all important qualities that likely make for a successful career as a surgeon and a FD. However, these traits are subjective in nature and are not easily measured. Nevertheless, this analysis provides an accurate cross-sectional representation of the current and past trends of the orthopaedic musculoskeletal oncology FDs currently serving as leaders at their respective institutions.

## Conclusion

To our knowledge, this is the first study that aims to define the common characteristics present in FDs of US accredited Musculoskeletal Oncology Fellowships in orthopaedic surgery. This study shows that orthopaedic musculoskeletal oncology FDs were mainly White (84.2%), male (78.9%), and in their late 40s; have no additional advanced degrees (73.7%); and have filled their role as FD for an average of 7.1 years. In addition, the mean Scopus H-index across all 19 FDs was 21.6, with 57.9% of FDs having an H-index of over 15 which is above average compared with FDs for other orthopaedic surgery fellowships. Also, these FDs seemed to have trained at a more diverse set of institutions compared with FDs in other orthopaedic surgery fellowships. Overall, these findings present objective data that may be helpful for future aspiring FDs in this field.
